# Occurrence and functional significance of the transcriptome in bovine (*Bos taurus*) spermatozoa

**DOI:** 10.1038/srep42392

**Published:** 2017-03-09

**Authors:** Sellappan Selvaraju, Sivashanmugam Parthipan, Lakshminarayana Somashekar, Atul P Kolte, B. Krishnan Binsila, Arunachalam Arangasamy, Janivara Parameshwaraiah Ravindra

**Affiliations:** 1 Reproductive Physiology Laboratory, Animal Physiology Division, ICAR- National Institute of Animal Nutrition and Physiology, Adugodi, Bengaluru-560030, India; 2 Omics Laboratory, Animal Nutrition Division, ICAR-National Institute of Animal Nutrition and Physiology, Adugodi, Bengaluru-560030, India.

## Abstract

Mammalian spermatozoa deliver various classes of RNAs to the oocyte during fertilization, and many of them may regulate fertility. The objective of the present study was to determine the composition and abundance of spermatozoal transcripts in fresh bull semen. The entire transcriptome of the spermatozoa from bulls (n = 3) was sequenced using two different platforms (Ion Proton and Illumina) to identify the maximum number of genes present in the spermatozoa. The bovine spermatozoa contained transcripts for 13,833 genes (transcripts per million, TPM > 10). Both intact and fragmented transcripts were found. These spermatozoal transcripts were associated with various stages of spermatogenesis, spermatozoal function, fertilization, and embryo development. The presence of intact transcripts of pregnancy-associated glycoproteins (*PAG*s) in the spermatozoa suggest a possible influence of sperm transcripts beyond early embryonic development. The specific regions (exon, intron, and exon-intron) of the particular spermatozoal transcripts might help regulate fertilization. This study demonstrates that the use of two different RNA-seq platforms provides a comprehensive profile of bovine spermatozoal RNA. Spermatozoal RNA profiling may be useful as a non-invasive method to delineate possible causes of male infertility and to predict fertility in a manner that is more effective than the conventional methods.

The mammalian spermatozoon transfers coding as well as non-coding transcripts to the oocyte during fertilization^1^. Spermatozoal transcript composition and expression levels are associated with spermatogenesis^2^, functional parameters of spermatozoa^3^, early embryonic development^4^,^5^ and pregnancy outcomes^6^,^7^. Spermatozoal transcripts also influence the phenotype of the offspring^8^ and might help in diagnosing and managing male infertility^5^,^9^. As species-specific variations can be expected, it is necessary to document the composition of the spermatozoal transcripts for each species to understand the factors associated with spermatogenesis, failed fertilizations and embryonic mortality and failures in interspecies hybrid reproductive processes, somatic cell cloning and other reproduction-related manipulations.

The spermatozoa contain both intact and biologically degraded RNAs in such low quantities that conventional techniques such as qPCR^10^,^11^, microarray^12^ and hybridization techniques^13^ may not provide complete information on the spermatozoal RNA composition and actual expression levels. The whole-transcriptome sequencing technique provides complete transcript information, including transcript variants, single nucleotide polymorphisms and novel transcripts^2^, in a cell or tissue and can be obtained from a relatively small amount of RNA within a reasonable time. Hence, this technique is appropriate to study the composition of spermatozoal transcripts.

Whole-transcriptome sequencing has been employed for assessing male fertility in humans^5^,^14^ and laboratory animals^15^. To date, only one report is available on the spermatozoal RNA composition of frozen bovine semen^16^. Because the composition and abundance of spermatozoal RNAs can be influenced by storage conditions^17^ and freeze-thaw procedures^18^, spermatozoal transcript quality and composition need to be studied in fresh semen. As the library preparation methods^14^ and sequencing platforms used also influence the outcome of sequencing results, the transcript profiles from different platforms need to be pooled to obtain complete transcript profiles. So far, no study has reported on whole-transcriptome sequencing of fresh spermatozoa using different platforms to explore the complete transcript composition and transcript expression levels in domestic animals. The objective of the present study was to determine the composition and abundance of spermatozoal transcripts in fresh bull semen using two different platforms to elucidate the possible molecular and functional association of the transcriptome with spermatogenesis, spermatozoal function, fertilization and embryonic development.

## Results

### Spermatozoal RNA yield and quality

**Figure 1:**
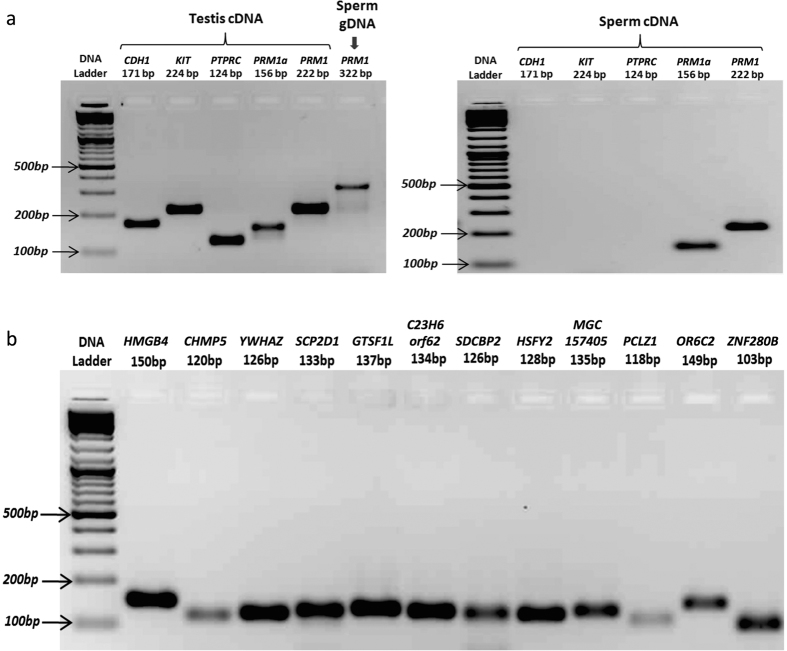
RNA isolation, quality control and validation of the presence of transcripts in bull spermatozoa. (**a**) The cDNA was synthesized from testis RNA amplified all the primers with desired product size, whereas the cDNA synthesized from the spermatozoal RNA amplified only for spermatozoa specific primer and failed to amplify other cell specific primers indicating the purity of the spermatozoal RNA from other contaminating cells and spermatozoal genomic DNA. (**b**) The transcripts identified in the RNA-seq platforms were validated by qPCR. The cDNA was synthesized using Superscript-II (Invitrogen, USA) and 10 ng (based on total RNA concentration used for cDNA synthesis) of cDNA was used for qPCR reaction. The PCR products were visualized in 1.8% agarose gel electrophoresis.

The spermatozoon contained 20–30 fg of RNA and these RNAs were fragmented, with a peak fragmentation size of 75 bp. RNA from the testis had intact 18 S and 28 S ribosomal RNAs (rRNAs), whereas the spermatozoa lacked any of rRNA peaks in the Bioanalyzer profile (Agilent, USA). The total RNA isolated from the spermatozoa was ensured to be free of gDNA and other RNA contaminants from somatic cells, leucocytes and germ cells. Testicular RNA and spermatozoal gDNA were used as positive controls (Fig. 1a).

### Characteristics of libraries, reads, and mapping statistics

**Figure 2:**
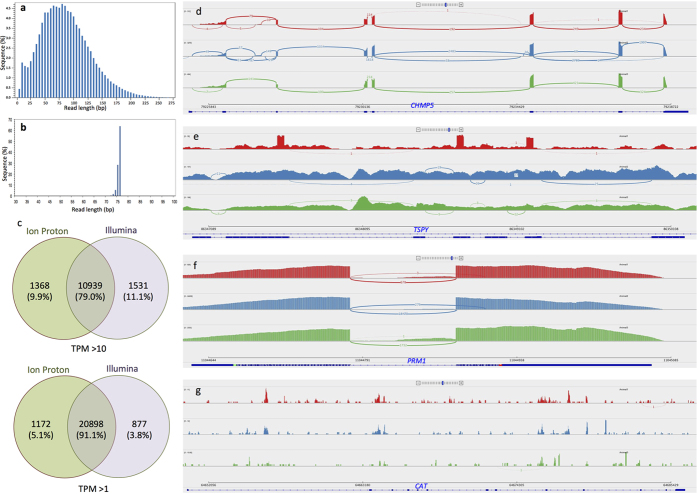
The read length distribution and total transcripts identified in both the platforms and read coverage nature of the spermatozoal transcripts. The read length distribution in (**a**) Ion Proton and (**b**) Illumina was analyzed by Bioanalyzer. The read length distribution differed significantly (p < 0.05) between platforms. (**c**) The total common genes identified in between the platforms were 10939 (TPM > 10) and 20898 (TPM > 1). The abundant transcripts were visually analyzed for reads coverage and observed that, the spermatozoal transcripts were exist in varying its nature/status. The spermatozoa contain (**d**) mature full length mRNA, (**e**) immature mRNA, (**f**) only exonic reads and (**g**) only intronic reads.

The isolated RNA was subjected to two different library preparation procedures. A single-end RNA sequencing library without size selection was prepared for Ion Proton (Ion Proton, Life Technologies, USA) analysis, and a paired-end RNA sequencing library with size selection was prepared for Illumina platform (Illumina Inc, USA) analysis. The constructed libraries were subjected to sequencing on the respective platforms and generated approximately 20 million reads/sample. The 95% of the reads generated from the Ion Proton and Illumina platform were between 10–130 bp (Fig. 2a), and 74–76 bp (Fig. 2b), respectively. The number of reads (mean ± SD, in millions) obtained from the Ion Proton and Illumina platforms after trimming were 19.2 ± 2.2 and 17.0 ± 1.9, respectively. The mapping percentages of reads from the Ion Proton and Illumina platforms were 54.6 ± 0.6 and 11.74 ± 3.2, respectively.

### Total number of transcripts and their expression level

The spermatozoa contained 12,307 and 12,470 genes (>10 TPM) based on the Ion Proton and Illumina platform analyses, respectively (Fig. 2c), and of these, 1,386 genes were highly abundant (TPM > 100). Approximately 11,405 genes were poorly expressed (TPM 1 to 10). The common genes observed between platforms were 10939 (79%) with TPM > 10 and 20898 (91%) with TPM > 1 (Fig. 2c). The PCR validation of the randomly selected transcripts confirmed the presence of spermatozoal mRNAs (Fig. 1b).

### Nature of spermatozoal transcripts

**Figure 3:**
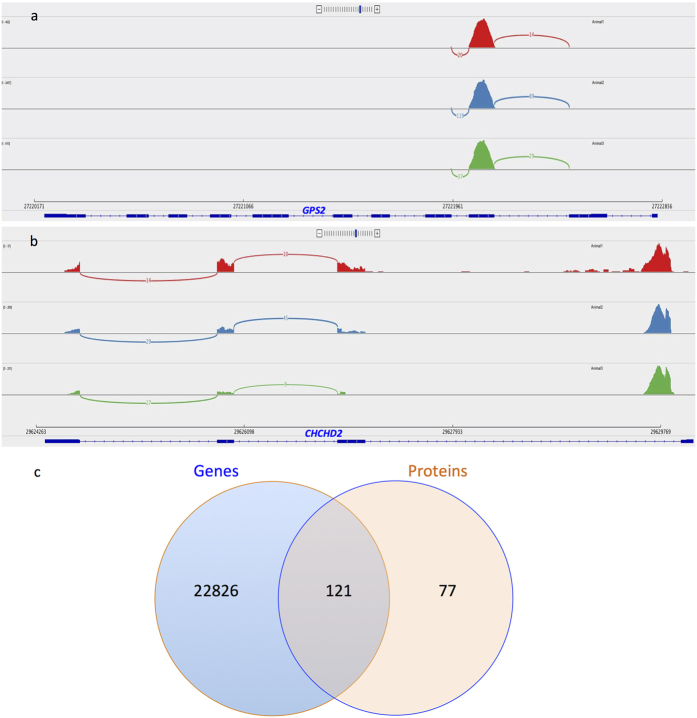
The spermatozoal RNAs retained specific regions/sequences for particular transcripts may influence fertility. (**a**) The GPS2 transcripts had only exon retained region, where as (**b**) *CHCHD2* had 3′ intronic retained region. Such RNA retained regions were consistently observed in all bulls and could be of fertility predictive value. (**c**) In order to get significant protein identities, genes (TPM > 1) obtained from the RNA-Seq were compared with the proteome data from LC-MS/MS (proteins with a minimum of 1 peptide per protein) and was observed that more than 61% of the proteins had corresponding transcripts in the bovine spermatozoa.

Spermatozoa carry intact mature mRNA (Fig. 2d), immature mRNA (Fig. 2e), and transcripts with only exonic reads (Fig. 2f) or only intronic reads (Fig. 2g). The analysis of spermatozoal transcript integrity revealed that approximately 36% of the top 100 transcripts selected based on TPM were full length and had intact 5′ and 3′ ends (Supplemental Table S1). The transcripts *PRM1*, *CHMP5* and *YWHAZ* were among the most abundant (Table 1) and are involved in male gamete generation and sperm function (Supplemental Table S2). It was also observed that spermatozoa consistently retained specific regions of particular transcripts in all animals with high read coverage. These retained regions could be either exonic or intronic. For example, the *GPS2* transcript retained its third exon with the high read coverage (Fig. 3a), whereas the *CHCHD2* transcript retained its 3′ intron sequence (Fig. 3b).

Spermatozoa carry highly abundant unannotated transcripts (LOCs, Supplemental Table S3), transfer RNAs (tRNAs), such as *TRNA-UAG-3* and *TRNAG-UCC-19* (Table 2), and lncRNAs, such as NONBTAT002138.2 and NONBTAT029740.1 (Supplemental Table S4). The miRNAs, *MIR196B* and *MIR365–2*, were abundant in the bovine spermatozoa (Table 2).

### Functions of spermatozoal transcripts

The abundant (TPM > 100) spermatozoal transcripts were significantly associated with germ cell development (p = 4.04E-03) (Supplemental Data 1 A). The transcripts influencing spermiogenesis were functionally associated with processes such as DNA packaging (p = 1.76E-03), chromatin assembly (p = 6.15E-03), chromatin silencing (1.49E-0.02), and DNA metabolic processes (p = 2.83E-0.02, *DNAJC2* and *TNP1*), which are essential for sperm nuclear formation. The spermatozoa also contained transcripts associated with cytoskeletal organization (p = 4.4E-02, *PICK1* and *PFN1*). Transcripts associated with biological processes such as the regulation of amide (p = 2.35E-06) and peptide (p = 3.69E-06) biosynthesis, translation (p = 4.61E-06), energy homeostasis and the generation of precursor metabolites (p = 6.09E-04, *FBP1* and *TRAP1*), motility (*CATSPER3*, TPM = 367) and acrosomal reactions (*PLCB1*, TPM = 7) were significantly enriched in the spermatozoa.

The spermatozoa contained transcripts (p = 2.2E-02) associated with reproductive processes (p = 3.87E-0.02) such as sperm-egg recognition (*PRSS37*, TPM = 137), the binding of spermatozoa to the zona pellucida (*HSPA1L*, TPM = 74) and egg activation (*PLCZ1*, TPM = 114) (Supplemental Data 1B). In addition, the spermatozoa carried many transcripts that might regulate different stages of embryogenesis, such as blastocyst development (p = 5.95E-03) and embryonic morphogenesis (p = 6.61E-03). The embryogenesis-associated transcripts were highly abundant (TPM > 300), and transcripts such as *BCL2L11* and *BRCA1* existed as intact and full length molecules. The molecular functions of the highly abundant spermatozoal transcripts are associated with structural components of ribosomes (p = 1.04E-07), whereas the less abundant transcripts are associated with ion transporter activity (Table 3), and these transcripts are constituents of cytosolic ribosomes (p = 5.61E-09) and main axon (p = 1.12E-03) (Table 3).

### Tissues and pathways

The spermatozoal transcripts were found in various tissues (Table 4). The expression levels of these highly abundant transcripts were significant in the placenta (p = 4.40E-04) and testes (p = 6.1E-02). The transcripts were found to be abundant in spermatids (p = 2.83E-08) and testicular tissue (p = 2.28E-06) (Table 4). Placenta-associated genes, such as *THSD4*, *PAG5*, *PAG7* and *PAG10*, were abundantly transcribed in spermatozoa, suggesting that these spermatozoal transcripts might play a role in implantation and placental development (Table 5). The abundantly expressed spermatozoal transcripts are associated with signaling pathways that are mainly involved in axon guidance (p = 0.01, Supplemental Figure S1a) and oocyte meiosis (p = 0.07, Supplemental Figure S1b) (Supplemental Table S5).

### Transcripts enriched in spermatozoa

The transcripts enriched in spermatozoa were identified by comparing the bull spermatozoal transcripts (TPM > 10) with previously reported human spermatozoal transcripts^19^. Although all the transcripts were not identified in both the bovine and human profiles, the expression of common transcripts specific to sperm, testes and extracellular seminal fluid were identified (Table 6). Some of the transcripts were also expressed in more than one cell/tissue types. The common transcripts present in all the animals in the present study were involved in metabolism, metal binding, motility and calcium signaling. The proteins involved in this second most top cluster are cold shock proteins and nucleic acid binding nature (Supplemental Table S6).

### Comparison of spermatozoal transcripts and protein profiles

The sperm protein profile characterized via LC-MS/MS revealed 198 proteins (Supplemental Data 1 C), 121 of which were found to have corresponding transcripts (>1 TPM) in the RNA-seq data (Fig. 3c). The respective proteins for a large number of transcripts (11,799 genes) were not found. The translated proteins play a significant (p < 0.05) role in metabolic functions (p = 1.4E-06, *GAPDHS* and *ENO1*), capacitation (p = 7.70E-02, *BSP3* and *PRKACA*), and events associated with fertilization (p = 1.40E-06, *BSP5* and *SPADH2*) (Table 7). The major pathway associations for the translated transcripts are in the categories of metabolic pathways, signaling cascades and oocyte meiosis (Supplemental Data 1D).

## Discussion

Existing spermiogram tests are insufficient to accurately assess male fertility; thus, research is being focused on identifying novel molecular markers that can be incorporated into the existing tests for the best possible accuracy. In this regard, spermatozoal transcriptomes have been investigated to assess male fertility^14^ and the success of assisted reproductive technologies^5^. So far, the spermatozoal transcript profiling has been carried out in humans^14^, bovines^16^, horses^20^ and mice^15^ to assess the role of spermatozoal RNA in fertility. Because cryopreservation affects the quality and quantity of spermatozoal RNA^18^, in the current study, we used fresh semen to examine the composition, abundance and possible functions of spermatozoal RNA. The comparison analyses of abundant top 328 transcripts revealed major variations between the studies (Supplemental Figure S3). Apart from cryopreservation procedure, the observed variation could also be due to RNA isolation methods, library preparation procedures, sequencing chemistry and analyses pipeline.

Each bovine spermatozoon contained 20–30 fg of fragmented RNA, with intact or biologically degraded ribosomal RNA^21^,^22^, which was evidenced by the absence of 28 S and 18 S peaks in the Bioanalyzer profile. The result indicates that bovine spermatozoa carry rRNAs that are either intact (*RPL23*, *RPL27A* and *RPS18*) or degraded (*RPL37, RPL36AL* and *RPL6*) and might be packaged into the cells during spermatogenesis^12^,^23^.

Also, because the library preparation methods and sequencing platforms used influence gene profiles and expression levels^14^, the current study used two different RNA-seq platforms, Ion Proton and Illumina, to identify the maximum number of genes in spermatozoa. Accordingly, the Ion Proton and Illumina analyses together identified 10,939 common genes (TPM > 10). Variations in library preparation methods and sequencing principles led to differences in the read length distributions and mapping percentages between the two platforms. The Ion Proton library preparation includes a ligase-enhanced genome detection method, which captures complete RNA profiles, including fragmented RNA, without size selection. By contrast, the Illumina Ultra-Low RNA Input Kit for cDNA library preparation uses size selection, a switching mechanism at the 5′ end of the RNA template, and increases the representation of GC-rich genes with low representation in rRNA. The Ion Proton and Illumina platforms employed single-end and paired- end reads, respectively. Hence, by exploiting the advantages of these two different platforms, comprehensive transcripts profiles were obtained in the present study. However we observed 79% common transcripts with TPM > 10 and 91% common transcripts with TPM > 1.The study established that both platforms are equally effective for profiling sperm transcriptome.

Bull spermatozoa contained transcripts for 13,838 genes, a number that is comparable with the findings of earlier reports in bovines^16^ and other species^12^,^14^,^15^. The reads mapping to the introns and exons were 25–30% and 6–8%, respectively, which confirms the presence of both immature and mature mRNAs in spermatozoa. The most abundant transcripts are expressed in the testes, epididymis, prostate gland and seminal vesicles. The transcripts associated with histone-to-protamine replacements, DNA repair, chromatin remodeling and epigenetic imprinting were abundant in the spermatozoa, indicating that a sudden burst of transcription might occur during spermatid development^12^,^14^.

The majority of the abundant spermatozoal transcripts were intact, and these transcripts might play a role in sperm function, fertilization events and early zygote development upon their transfer to the oocytes^1^,^13^,^14^,^24^. One such abundant transcript, *YWHAZ*, observed in the present study has been reported to regulate spermatogenesis and acrosomal reactions^25^. Furthermore, *YWHAZ* gene-knockout males have been found to be infertile^26^. Two other transcripts, *KIF5C* and *KCNJ6*, found to be abundant in spermatozoa are involved in microtubule function. Such transcripts are also abundant in the cerebral cortex^27^,^28^. The presence of such brain-associated transcripts in spermatozoa has been previously suggested, but the functional relevance of this result is not clearly understood^9^. LOCs were abundant in the spermatozoal RNA profile, as reported earlier^16^, and characterizing these transcripts and their relevant proteins may elucidate the role of these genes in fertilization and embryo development.

Apart from these findings, studies have suggested that sperm cells provide noncoding RNA (ncRNA)^24^ and miRNAs^29^ that may influence semen fertility. One such class of ncRNA, tRNAs, were abundant in bovine spermatozoa. The tRNA composition and their function in bovine spermatozoa have not been completely studied, but a previous report revealed that tRNA-derived small RNAs (tsRNAs) are delivered to the oocyte and utilized during early embryonic development in mice^30^. Spermatozoal tsRNAs have been reported to affect embryonic gene expression via transcriptional cascade change in the early embryo and alter offspring phenotype in mouse^31^. Such elements may play a role in the confrontation and consolidation of events during the process of fertilization for successful embryo development^5^,^8^,^9^,^32^. Another ncRNA, miRNA, is also a potential regulator of semen fertility. The second most abundant miRNA observed in the present study, *miR-365-2*, is known to have *BCL2* as one of its targets^33^, and the suppression of *BCL2* is essential for the progression of a fertilized oocyte into the cleavage stage; therefore sperm-derived factors are thought to be essential for the development of preimplantation embryos^34^. Although a role of long non-coding RNA (lncRNA) on spermatogenesis has been reported^35^, further studies are essential in bovine to better understand the roles of ncRNAs on spermatozoa fertility.

Spermatozoal transcripts are significantly over represented in spermatids, indicating that many of the spermatozoal transcripts are related to sperm function^12^,^14^. The *TNP1*, *RAD21*, *UBE2B* and *RAN* transcripts were highly abundant (TPM > 100) in spermatozoa and are involved in the crucial stage of spermatid development, wherein high-order chromosomal organization takes place. *RAD21* is expressed in spermatogonia^36^ and is involved in sister chromatid cohesion and segregation during meiosis^36^,^37^,^38^. Mice lacking *RAD21* had been found to have gonadal atrophy and infertility^39^. Similarly, *RAN*, in association with calcineurin, regulates spermatogenic meiosis^40^, and the absence of RAN binding protein 1 (*RNABP1*) might result in male infertility^41^.

The presence of abundant nuclear-encoded transcripts/proteins that are essential for the condensation of nuclear material in spermatozoa (chromosome segregation, DNA repair, chromatin remodeling and chromatin condensation) was observed in the study. These transcripts help determine the ability of spermatozoa to fertilize the egg, and any alterations in the expressions of these nuclear-encoded transcripts might lead to male infertility^42^. After the nuclear organization of the chromosomes, spermatids start to elongate to form their typical cytoskeletal structure. The spermatozoa carry high numbers of transcripts associated with cytoskeleton organization. For example, *TEKT1* is important for spermatozoal cytoskeleton development, and this protein is localized in the apical region of the acrosome and on flagella and could play a key role during fertilization^43^. Similarly, thymosin ß10 and CAPZA3 are known to be involved in spermatozoa capacitation and spermatozoa egg fusion^44^,^45^. The proper organization of the centrosome in spermatozoa is essential for oocyte genome activation leading to successful zygote formation, and any defects in this process might lead to male infertility^46^. In the present study, transcripts (*MAP7*, *PTK2*, *PLK1S1*, *MYH9* and *PRKCZ*) involved in centrosome organization were observed in the ejaculated spermatozoa, suggesting that the presence of these transcripts might reflect events associated with spermatogenesis and sperm function. The presence of motility-associated transcripts, such as *CATSPER* and *SPAG*, indicate the fertility of the male^47^,^48^, and a knockout study has shown that *SPAG* genes are also essential for the structural integrity of spermatozoa^48^.

Genes such as *PLCZ1* and *PLCB1* are involved in calcium signaling and the spermatozoon-induced activation of an oocyte leading to its final maturation^49^,^50^. The abundant spermatozoon-specific transcript *PLCZ1* interacts with phosphatidylinositol and thus activates the process of oocyte maturation via Ca^2+^oscillations^49^. The finding that spermatozoa also synthesize proteins necessary in the female reproductive system during the acrosomal reaction^51^,^52^ opens a window to understanding the possible role of these proteins during and after fertilization.

Spermatozoa carry fertilin transcripts (*ADAM1B*, *ADAM2* and *ADAM32*) along with the corresponding proteins. These proteins regulate sperm motility in the reproductive tract and sperm-zona pellucida binding and penetration^52^,^53^,^54^. Thus, an absence of these proteins might affect fertility^55^. Spermatozoal motility is correlated with mitochondrial function^56^, which regulates energy generation, apoptosis and calcium homeostasis. In this study, *COX5 A* and *COXI1* transcripts, which are associated with mitochondrial function, were observed in the spermatozoa along with their respective proteins. The altered expression of these genes might cause mitochondrial dysfunction and impaired fertility^57^.

This study confirms the existence of embryogenesis-associated transcripts, such as *ZP3* and *FOXG1*, in spermatozoa^58^, and some of these transcripts were observed to be full length and intact. The abundance of these transcripts was also high (TPM 10–100), and they are likely delivered to the oocyte where they may act as templates for protein synthesis by the maternal translational machinery upon activation of the oocyte genome^4^. In the present study, we observed that *BCL2L11* and *BRCA1* transcripts were abundant (TPM 300–700) in spermatozoa. The expression of *BCL2L11* by cumulus cells can be used to assess the status of the embryo quality in humans^59^, but the role of these genes on male fertility needs to be established.

Studies conducted so far suggest that the spermatozoal transcripts have a role in fertilization and early embryonic development. However, the placental abnormalities reported from somatic cell nuclear transfer studies^60^,^61^ indicate that such abnormalities could also be due the absence of the paternal contribution. In the present study, placental development-associated transcripts, such as *PAG5*, *PAG7* and *PAG10*, were observed to be full length and intact in spermatozoa, suggesting that sperm-borne transcripts have an influence beyond early embryonic development, such as during the processes of implantation and placentation.

The functional annotation of the common transcripts observed in animals revealed that these transcripts are involved in metabolism, motility and translation regulation. In mouse, cold shock protein with nucleic acids binding properties, *YBX2* regulates male fertility^62^ and *YBX1* is essential for survival of embryos^63^. These proteins have been reported to stabilize cytoplasmic RNA and suppression of translation of germ cell mRNAs. Presence of such clusters of transcripts in spermatozoa may be considered as a marker of semen quality.

Enriched spermatozoal transcripts and protein pathways associated with axon guidance and oocyte meiosis were identified, further suggesting that these transcripts might have a potential role in sperm function and fertilization. Similarly, the spermatozoal proteins produced from their respective transcripts might play a role in sperm metabolism, oocyte meiosis and fertilization. The occurrence of functional transcripts in the spermatozoa indicate their significant role in regulating spermatogenesis and embryogenesis.

Studies have confirmed the presence of many RNAs in mature spermatozoa from different species^3^,^14^,^15^,^16^, but the role of very few of these transcripts are correlated with spermatozoal function and fertilization^12^,^13^. To the best of our knowledge, although studies have suggested roles for spermatozoal transcripts in various facets of fertilization and up to early embryonic development^4^,^5^, no clear experimental evidence is available on the influence of these transcripts beyond the activation of the embryonic genome.

## Conclusion

Bull spermatozoa contain many functional genes, and several of these genes have functions associated with different stages of spermatogenesis, spermatozoa kinetics, fertilization and embryonic development. As the mRNAs are short lived, many of the proposed functions of the spermatozoal transcripts beyond early embryonic development are speculative and need to be proved experimentally. The findings from the present study provide two different insights. The first is that spermatozoal carry remnant mRNAs along with the corresponding proteins, which are retrospectively indicative of successful spermatogenic events. Second, spermatozoal contain the transcripts that might play vital roles in fertilization and embryogenesis. Spermatozoa transcriptome profiling could be a non-invasive approach for the identification of transcripts that are indicative of sperm fertility.

## Materials and Methods

### Sample collection

Semen samples from bulls (n = 3) that were known to be fertile were obtained from Nandini Spermatozoa Station (Karnataka Milk Federation, ISO 9001:2008 certified unit, Hessarghatta, Bengaluru, India). All experiments were performed in accordance with the relevant guidelines and regulations. The experimental protocols were approved by the Institute Research Committee, ICAR-NIANP, Bengaluru and Task Force (Animal Biotechnology), Department of Biotechnology, Government of India. From each bull, four ejaculates were pooled to minimize transcript expression profile biases. The pooled semen samples of individual bulls were subjected to RNA isolation and to whole-transcriptome sequencing using Ion Proton and Illumina (NextSeq-500) platforms.

### Spermatozoa preparation for RNA isolation

The semen samples were purified using a 50% Bovipure gradient solution (Nidacon, Sweden) in phosphate-buffered saline (PBS, pH 7.2). Then, 1 ml of each semen sample was slowly layered on top of 4 mL of 50% Bovipure solution in 15 mL conical-bottom centrifuge tubes. The samples were centrifuged at 200 *g* at room temperature (28 °C) for 20 min. After centrifugation, the gradient solutions were removed without disturbing the spermatozoal pellet. The spermatozoal pellet was re-suspended in 10 mL of PBS and centrifuged at 2500 *g* for 5 min at 4 °C. It was then re-suspended in 1 mL of PBS, and spermatozoon concentrations were measured using a hemocytometer.

### Total RNA isolation, quantification and qualification

Total RNA was isolated as described earlier^22^. In brief, the spermatozoon samples (30–40 × 10^6^ cells) were homogenized and lysed with a lysis buffer cocktail (0.5 mL of lysis buffer from the kit + 0.5 mL of TRIzol), followed by phase separation with 0.2 mL of chloroform. The remaining protocol was performed following the manufacturer’s instructions (PureLink RNA mini kit, Ambion, USA). The isolated RNA was treated with DNase (TURBO DNase free, Ambion, USA) to eliminate traces of gDNA. Total RNA was quantified using a fluorometer (Qubit 2.0, Invitrogen, USA) with an RNA high-sensitivity assay kit (Invitrogen, USA), and RNA quality was analyzed using a spectrophotometer to check for contamination from proteins and organic solvents. The Bioanalyzer system was used to assess the fragment size distribution of spermatozoal RNA. After quality and quantity analyses, total RNA samples were converted into cDNA using Superscript II (Invitrogen, USA). qPCR experiments were carried out to check for contamination with an input RNA concentration of 10 ng/reaction using cell-specific primers for RNA from other cell types and intron-spanning primers to detect spermatozoal gDNA contamination (Supplemental Table S7). The qPCR analyses were performed based on SYBR Green chemistry (MAXIMA SYBR Green Master Mix, Fermentas, USA).

### Library preparation and RNA-seq

#### Ion Proton platform

The library was prepared using an Ion Total RNA-Seq Kit v2 (Life technologies, USA) according to the manufacturer’s instructions, with slight modifications. During the library preparation, the fragmentation step was omitted due to the fragmented nature of spermatozoal RNA. Spermatozoal RNA was not enriched, and size selection was avoided. The template was prepared for sequencing using an Ion Express Template Kit (Life Technologies, USA). The constructed libraries were subjected to sequencing using the Ion Proton platform (Ion Proton™, Life Technologies, USA), generating 20 million reads/sample.

#### Illumina (N extSeq-500) platform

Total RNA was converted to double-stranded cDNA (ds cDNA) using a SMARTer Ultra-Low Input RNA Kit for the Illumina platform (Clontech Laboratories, CA, USA) followed by covaris shearing and end-repair of the overhangs resulting from the shearing. The paired-end RNA sequencing library was prepared using an Illumina TruSeq Total RNA Library Preparation Kit as described in the manufacturer’s protocol. The libraries were quantified using a DNA high-sensitivity assay kit and were sequenced using 2 × 75 paired-end (PE) chemistry using the NextSeq-500 platform, and approximately 20 million PE reads were generated for each sample.

#### Mapping of reads to the bovine genome

The initial quality of the raw reads was analyzed using FastQC (Babraham Bioinformatics, UK). After analyzing read quality (based on sequence quality scores, GC distribution of reads, sequence length distribution, sequence duplication level, adapter content and Kmer content), the adapter sequence, primers and low quality reads were trimmed to improve read quality and to reduce transcript expression profile biases. The reads (before and after trimming) were mapped to the bovine genome (Btau_4.6.1/Btau7) using the CLC Genomics Workbench (Version 8.0.1, CLC Bio, Qiagen, USA). Transcript expression levels were calculated based on TPM.

#### Transcripts classification and gene ontology

To determine the number of genes present in bull spermatozoa, the transcripts from both platforms were combined based on TPM. The highly abundant transcripts expressed in the spermatozoa were identified based on average TPM along with unique gene reads (>50 reads/transcript). The unique gene read parameter was included to make the selection process more stringent to ensure that the true abundance of the transcripts was identified. To assess the possible functions of spermatozoal RNAs, the common transcripts obtained from both RNA-seq platforms with TPM > 10 and >100 underwent gene ontology and functional annotation analyses.

#### Validation of RNA-seq data via qPCR

The transcripts were selected based on the RNA-seq data analysis to validate the sequence data (transcript presence, abundance, intactness and expression level) of spermatozoal RNA. The primers were designed for the selected transcripts (Supplemental Table S8) and validated using SYBR Green chemistry. Equal input template concentrations (10 ng/reaction) were used to validate the differences in the presence and expression of selected transcripts. Quantitative cycle values were used to calculate expression levels, and desired product sizes were confirmed via 1.8% agarose gel electrophoresis.

#### In-solution digestion of sperm proteins

To identify translated transcripts in spermatozoa, 22,948 genes (TPM > 1) from both platforms were compared with the sperm proteome obtained via LC-MS/MS. Protein samples from randomly selected spermatozoa from two bulls were precipitated using a mixture of acetone (50%), ethanol (50%) and acetic acid (0.1%) and incubated overnight at −20 °C. The obtained protein pellet was resuspended in 20% acetonitrile (ACN) in a 100 mM NH_4_HCO_3_ buffer. Protein concentrations were determined using a BCA assay (Sigma Aldrich, USA), and the pH of samples was adjusted to 8.5. For in-solution digestions, 20 μg of protein was dissolved in 20% ACN in a 100 mM NH_4_HCO_3_ buffer, followed by the reduction of the cysteine residues in the sample with the addition of 10 mM dithiothreitol (Pierce, Thermo Fisher Scientific, USA), and samples were then incubated at 56 °C for 30 min. Then, the samples were alkylated in the presence of 56 mM iodoacetamide (Pierce, Thermo Fisher Scientific, USA) and incubated in the dark for 30 min. Ice-cold trypsin (13 ng/μL) was added to the samples at a 1:20 ratio and mixed well. The tubes were incubated at 37 °C for 16 hours, and the tubes were then chilled on ice and very briefly centrifuged. Formic acid (5%) was then added to the tubes to stop the reaction, and the pH was adjusted to ~3.0. The digested peptides were vacuum dried, reconstituted in 15 μL of 2% ACN in 0.1% formic acid, and incubated with occasional vortexing for 30 min. The contents in the tubes were spun at 5000 rpm for 1 min, and the digested peptides were subjected to mass spectrometry analysis.

#### High resolution mass spectrometry analysis

The samples (1 μl, containing 600 ng of protein) were injected into an LC-MS/MS column and allowed to separate for 180 min in a RPLC gradient, followed by data acquisition with a Thermo LTQ Orbitrap Discovery system. The mass precision for the experiment was set to 4 ppm, with a S/N threshold of one. The minimum/maximum precursor mass range was 350–5000 Da, and the maximum collision energy was set to 1000 in an ESI-TRAP. The precursor mass tolerance was set to 10 ppm and fragment mass tolerance to 0.8 Da. The oxidation of methionine (M), carbamidomethylation of cysteine (C), and carboxymethyl (C), acetyl (N-term), Gln- > pyro-Glu (N-term Q) and Glu- > pyro-Glu (N-term E) alterations were considered as dynamic modifications. The target Decoy PSM validator was selected as the processing node with a target false discovery rate of not more than 0.01. The data were analyzed via a standard approach using MASCOT 2.4 as the search engine in Proteome Discoverer 1.4 (Centre for Cellular and Molecular Platforms, NCBS-TIFR, Bengaluru, India). The data were searched against the *Bos taurus* database (NCBI), and a minimum of two high-confidence peptide matches were considered a prerequisite for identifying the proteins. The proteome data obtained from two bulls were consolidated based on the criteria of a minimum of one peptide per protein to obtain significant protein identities for matching with sperm transcripts with >1 TPM.

#### Bioinformatics and statistical analysis

Total read counts/transcript were measured for 30,304 genes for each sample obtained from both RNA-seq methods. The TPM values were calculated, and comparisons were made between and within the RNA-seq platform results. The intactness of transcripts was analyzed by manually visualizing the read coverage of the top 100 transcripts selected based on their TPM. The intactness was assessed based on their 5′ and 3′ read coverage with unique gene reads (more than 5). The proportion-based Kal’s test (Z-test) was performed to identify the transcripts that differed significantly between the RNA-seq methods.

Gene ontology was analyzed with 20,689 annotations available in the bovine gene ontology consortium. The commonly expressed transcripts among the animals were identified irrespective of the platforms and the transcripts with non-significant difference between the platforms were selected and the subgroup of RNAs were identified. The ontological analysis (biological process, molecular functions, cellular components, pathways and tissue expression) for the transcripts with >10 TPM, commonly expressed subgroup of RNAs and the translated transcripts was performed using the online Database for Annotation, Visualization and Integrated Discovery (DAVID, v6.8), PANTHER (v10.0) and Gene Ranker (Genomatix software suite, v3.7) using *Bos taurus* as the background. Tissue enrichment associated with spermatozoal transcripts was analyzed using Gene Ranker (Genomatix software suite, v3.7) using *Homo sapiens* as the background. The statistical test for GO enrichment from the DAVID analysis was the Benjamini p value corrected for the false discovery rate, and for Gene Ranker, it was Fisher’s exact test. For all experiments, p values less than 0.05 were considered statistically significant.

## Additional Information

**How to cite this article**: Selvaraju, S. *et al*. Occurrence and functional significance of the transcriptome in bovine (*Bos taurus*) spermatozoa. *Sci. Rep.* **7**, 42392; doi: 10.1038/srep42392 (2017).

**Publisher's note:** Springer Nature remains neutral with regard to jurisdictional claims in published maps and institutional affiliations.

## Supplementary Material

Supplementary Information

Supplementary Dataset 1

## Figures and Tables

**Table 1:** The highly abundant (Top 20) spermatozoal transcripts identified based on the TPM (average of all the bulls in both the RNA-seq platforms) and unique gene reads (UGR).

Gene ID	Gene Name	TPM	UGR
*PRM1*	Protamine 1	8659	120
*YWHAZ*	Tyrosine 3-monooxygenase/tryptophan 5-monooxygenase activation protein, zeta	3050	84
*FABP1*	Fatty acid binding protein 1	2923	1074
*SCP2D1*	Sterol-binding domain containing 1	2726	182
*THSD4*	Thrombospondin type 1 domain containing 4	1961	2506
*CHMP5*	Charged multivesicular body protein 5	1693	260
*NR2E3*	Nuclear receptor subfamily 2 group E member 3	1610	1241
*SV2C*	Synaptic vesicle glycoprotein 2 C	1518	2592
*MGC137055*	Det1 and ddb1 associated 1	1434	74
*GTSF1L*	Gametocyte specific factor 1-like	1416	155
*TOE1*	Target of EGR1, member 1 (nuclear)	1359	1743
*SLC16A7*	Solute carrier family 16 member 7	1284	2831
*MCOLN2*	Mucolipin 2	1231	1756
*UNC119*	Unc-119 lipid binding chaperone	1136	790
*CXCR4*	C-X-C motif chemokine receptor 4	1095	975
*PAG5*	Pregnancy-associated glycoprotein 5	971	962
*MMP2*	Matrix metallopeptidase 2 (gelatinase A, 72 kDa gelatinase, 72 kDa type IV collagenase)	933	1417
*ITPA*	Inosine triphosphatase (nucleoside triphosphate pyrophosphatase)	919	458
*CCDC181*	Coiled-coil domain containing 181	919	144
*DNAJB12*	DNAJ heat shock protein family (Hsp40) member B12	914	2315

**Table 2:** The abundant transfer RNAs (tRNAs) and the microRNAs (miRNAs) identified in bovine spermatozoa.

Gene ID	TPM	UGR
**tRNA**		
*TRNAL-UAG_3*	25756	67
*TRNAG-UCC_19*	19177	1719
*TRNAD-GUC_11*	13905	850
*TRNAE-UUC_6*	13366	844
*TRNAE-UUC_90*	12932	0
*TRNAE-UUC_60*	12870	0
*TRNAG-UCC_49*	12555	1
*TRNAL-AAG_1*	10344	940
*TRNAG-GCC_4*	4736	0
*TRNAG-GCC_6*	4662	0
miRNA*		
*MIR196B*	597	18
*MIR365-2*	322	0
*MIR2478*	184	7
*MIR2455*	136	4
*MIR130A*	134	4
*MIRLET7F-1*	119	4
*MIR411*	69	2
*MIR23A*	65	2
*MIR1197*	56	3
*MIR1193*	55	8

These transcripts abundance were obtained by averaging the TPM of all the bulls in both the RNA-seq platforms. *For miRNA analysis only the transcripts from Ion–Proton platform was used.

**Table 3:** The molecular functions and cellular localization of spermatozoal transcripts obtained from functional annotation analysis (Gene Ranker, Genomatix and DAVID bioinformatics) based on their transcription level (TPM).

GO-Term	P-Value	Genes Observed
Molecular function		
TPM > 100		
Structural constituent of ribosome	1.04E-07	24
Structural molecule activity	1.88E-04	29
Poly(A) RNA binding	7.33E-03	29
Ribonuclease A activity	8.24E-03	2
Endoribonuclease activity, producing 3′-Phosphomonoesters	8.24E-03	2
mRNA binding	1.01E-02	6
Copper ion binding	1.14E-02	5
Arp2/3 complex binding	1.62E-02	2
Photoreceptor activity	1.62E-02	2
TPM > 10		
Metal ion transmembrane transporter activity	1.32E-04	55
Ion transmembrane transporter activity	4.48E-04	130
Neurotransmitter:sodium symporter activity	5.38E-04	9
Substrate-specific transmembrane transporter activity	5.73E-04	141
Solute:sodium symporter activity	7.06E-04	13
Transmembrane transporter activity	7.57E-04	148
Structural molecule activity	8.71E-04	141
G-protein coupled amine receptor activity	1.45E-03	10
Structural constituent of ribosome	1.48E-03	75
Signaling receptor activity	1.75E-03	103
**Cellular component**		
TPM > 100		
Cytosolic ribosome	5.61E-09	19
Ribosomal subunit	3.66E-08	23
Ribosome	9.16E-08	27
Cytosolic large ribosomal subunit	1.61E-07	13
Cytosolic part	5.45E-07	21
Large ribosomal subunit	2.13E-05	13
Ribonucleoprotein complex	1.91E-04	33
Intracellular non-membrane-bounded organelle	4.38E-04	85
Non-membrane-bounded organelle	4.38E-04	85
Small ribosomal subunit	4.98E-04	10
TPM > 10		
Cytosolic ribosome	2.67E-06	52
Cytosolic part	2.85E-05	72
Integral component of plasma membrane	9.70E-05	102
Intrinsic component of plasma membrane	2.89E-04	105
Cytosolic small ribosomal subunit	3.00E-04	22
Cytosolic large ribosomal subunit	9.23E-04	29
Ribosomal subunit	1.01E-03	68
Main axon	1.12E-03	8
Small ribosomal subunit	1.40E-03	35
Plasma membrane	1.65E-03	450

The analysis was carried out using *Bos taurus* as background.

**Table 4:** The tissue level association of spermatozoal transcripts.

Tissues	P-Value	Genes Observed
Tissue*		
Liver	1.50E-05	116
Placenta	4.40E-04	17
Ileum	2.30E-03	76
Spleen	2.40E-02	7
Kidney	2.70E-02	14
Lymphoid	3.30E-02	5
Testis	6.10E-02	35
Lymphoid epithelium	6.50E-02	10
Adrenal cortex	6.90E-02	4
Seminal vesicle	7.40E-02	3
**Tissue over represented^#^**		
Spermatid	2.83E-08	55
Testis	2.28E-06	103
Entire testis	3.86E-05	92
Adrenal gland sample	1.83E-04	4
Parietal lobe	3.63E-04	16
Posterior horn cells	3.65E-04	12
Bone and bones	5.02E-04	6
Structure of hepatic vein	5.07E-04	7
Substantia innominata	5.07E-04	7
Entire hepatic vein	5.07E-04	7

^*^The transcripts with more than 100 TPM were included for tissue expression analyses using *Bos taurus* as background in DAVID bioinformatics software.

^#^The transcripts with more than 100 TPM were included for tissue overrepresentation analyses using *Homo sapiens* as background in Gene Ranker (Genomatix, Qiagen, USA).

**Table 5:** The group of pregnancy-associated glycoprotein (*PAG*) identified in bovine spermatozoa.

Gene ID	TPM	UGR
*PAG5*	971	962
*PAG10*	440	416
*PAG7*	375	119
*PAG21*	302	193
*PAG18*	265	238
*PAG12*	255	372
*PAG2*	225	200
*PAG16*	145	143
*PAG1_2*	145	333
*PAG20*	144	233
*PAG15*	129	210
*PAG4*	104	221
*PAG3*	102	155
*PAG1_1*	36	183
*PAG11_1*	35	63
*PAG19*	16	0

These transcripts abundance were obtained by averaging the TPM of all the bulls in both the RNA-seq platforms.

**Table 6:** The bull spermatozoa enriched transcripts identified by comparing the transcripts (TPM, > 10) from the present study with human spermatozoal transcripts^19^.

Enriched tissue/cell	List of transcripts
Spermatozoa	*CCDC168, FAM186A, FAM209B, CAPZA3, GRID2, LTN1, PRSS37, ERICH2, FAM71B, KIAA1731, LRRD1, IFT43, CEP152, PPP2R2B, DNAJC9, ADAMTS6, RBBP6, CYB5R4, TMCO2, DHX36, KIF5C, PICK1, FAM71D, DCUN1D1, TTLL3, CABS1, SPATA7* and *H1FNT*
Testis	*SULF2, PRSS21, SMOC2, ADAM32, IQCF5, IQCF1, DYNLRB2, RAE1, MMP2, CD59, DKKL1, CADM1, ROPN1L, GSTM3, SIAE, PSMG1, SPA17* and *GOLGA4*
Extracellular (Seminal fluid)	*ZC3H15, PACSIN2, NUCB1, MRPS36, USMG5, SUMF2, BLVRB, DCTN4, SPOCK1, UNC13B, LSM2, MRPS23, DUSP3, SORD, SND1, THTPA, TIMMDC1, PPAP2A, YWHAZ* and *DSTN*
Spermatozoa + Testis	*BCAP29, TEX35, TMCO5A, CYLC2, HSPA4L, MLF1, HSPA1L, TSPAN16, ALMS1, SPATA6, NRBP1* and *HMGB4*
Spermatozoa + Extracellular	*TRA2A, HNRNPH1, UBTD2* and *SHOC2*
Testis + Extracellular	*CAPRIN1, MORN2, PSMG2, IGF1R, RAN, TMSB4X, WDTC1, COX5B, B2M, EEF1D, RPS19, RPL13A, RPS18, RPS3A, RPS12, RPL31, RPL27, RPLP0, RPL27A, RPS25, ATP5L, ACTB, RPL3, RPS14, RPS3, RPL18A* and *FTH1*
Spermatozoa + Testis + Extracellular	*GKAP1, DCAF5, TFAM, UBXN4, BPIFA3, VPRBP, EIF4G3, SPAG16, FXR1, DDX5, SKP1, CRISP2, TRA2B, NRD1, GSG1, CABYR, ODF2, OAZ3, AKAP12, LRTOMT, SPATA18, CHD2, ACSBG2, LPIN1, UBC, BRK1, CFL1, UBB, RPL23, UBA52, SMCP, RPS27A, RPS23, CHCHD3* and *TPT1*

**Table 7:** The functional annotation of the translated transcripts present in the spermatozoa.

Description	P-Value	Genes Observed
**Biological process**		
Glycolytic process	1.40E-06	6
Single fertilization	1.40E-06	6
Tricarboxylic acid cycle	1.70E-04	5
Substantia nigra development	1.60E-02	3
ATP synthesis coupled proton transport	2.30E-02	3
Hydrogen ion transmembrane transport	5.60E-02	3
Sperm capacitation	7.70E-02	2
Mitochondrial electron transport, cytochrome c to oxygen	7.70E-02	2
Nucleoside triphosphate biosynthetic process	8.90E-02	2
**Molecular function**		
Protein binding	4.00E-03	8
Protein domain specific binding	1.20E-02	4
Glucose binding	2.60E-02	2
Succinate-CoA ligase (GDP-forming) activity	2.60E-02	2
Phosphopyruvate hydratase activity	2.60E-02	2
Cytochrome-c oxidase activity	3.00E-02	3
ATP binding	3.30E-02	11
Protein kinase A catalytic subunit binding	6.40E-02	2
**Cellular component**		
Sperm fibrous sheath	5.40E-04	3
Extracellular exosome	7.50E-04	21
Myelin sheath	2.30E-03	6
Mitochondrion	4.90E-03	13
Mitochondrial matrix	1.20E-02	6
Acrosomal vesicle	1.90E-02	3
Extracellular space	2.40E-02	11
Mitochondrial proton-transporting ATP synthase complex	2.70E-02	3
Inner acrosomal membrane	2.70E-02	2
Phosphopyruvate hydratase complex	2.70E-02	2
Proton-transporting ATP synthase complex, catalytic core F(1)	4.00E-02	2
Plasma membrane raft	5.30E-02	2
Focal adhesion	5.90E-02	5
Sperm principal piece	6.60E-02	2
