# Supplementary material for: Occurrence and functional significance of the transcriptome in bovine (Bos taurus) spermatozoa
Source: Sci Rep. 2017 Mar 9;7:42392. doi: 10.1038/srep42392 (PMC5343582; doi:10.1038/srep42392)
Supplement: Supplementary Information [file srep42392-s1.pdf]

Supplementary information

Occurrence and functional significance of the transcriptome in bovine (*Bos taurus*) spermatozoa

Sellappan Selvaraju[1,*], Sivashanmugam Parthipan[1,#], Lakshminarayana Somashekar[1,#], Atul P Kolte[2], Binsila B Krishnan[1] , Arunachalam Arangasamy[1] and Janivara Parameshwaraiah Ravindra[1]

[1]Reproductive Physiology laboratory, Animal Physiology Division, [2]Omics laboratory, Animal Nutrition Division, ICAR-National Institute of Animal Nutrition and Physiology, Adugodi, Bengaluru-560030

[#]Department of Biochemistry, Jain University, Bengaluru-560001

Supplemental Table S1: The RNA integrity of top 100 abundant spermatozoal transcripts. The transcripts integrity was analyzed manually by visualizing the read coverage.

Gene ID	TPM	UGR	Intact*	5' coverage	3' coverage
PRM1	8659	120	Yes	Yes	Yes
YWHAZ	3050	84	Yes	Yes	Yes
SCP2D1	2726	182	Yes	Yes	Yes
CHMP5	1693	260	Yes	No	Yes
MGC137055	1434	74	Yes	No	Yes
GTSF1L	1416	155	Yes	No	Yes
PAG5	971	962	Yes	No	Yes
CCDC181	919	144	Yes	Yes	Yes
TNP1	875	308	Yes	Yes	Yes
CCDC85A	829	3481	Yes	No	Yes
C23H6orf62	788	207	Yes	Yes	Yes
BCL2L11	783	74	Yes	Yes	Yes
CCDC168	635	179	Yes	Yes	Yes
SSRP1	615	219	Yes	No	Yes
FAM209B	612	77	Yes	Yes	Yes
CAPZA3	558	183	Yes	Yes	Yes
NEK3	493	636	Yes	Yes	No
RAD21	474	242	Yes	Yes	Yes
SP2	461	390	Yes	No	No
SF3B1	456	361	Yes	Yes	Yes
TMED6	455	10	Yes	Yes	Yes
PPWD1	445	37	Yes	Yes	Yes
PAG10	440	416	Yes	No	No
INVS	439	2384	Yes	No	No
RPL23	424	22	Yes	No	Yes
KIAA1731	422	126	Yes	Yes	Yes
ODZ3	419	5074	Yes	No	No
RPL27A	407	536	Yes	No	Yes
LRRD1	407	27	Yes	Yes	Yes
RPS18	403	60	Yes	Yes	Yes
SFXN3	403	557	Yes	No	No
IFN-TAU	389	118	Yes	Yes	Yes

IGF1R	384	5979	Yes	No	Yes
SH3GL2	375	2332	Yes	No	No
PAG7	375	119	Yes	Yes	Yes
VPS4A	368	472	Yes	No	Yes
FABP1	2923	1074	No	Yes	No
THSD4	1961	2506	No	No	No
NR2E3	1610	1241	No	No	No
SV2C	1518	2592	No	No	No
TOE1	1359	1743	No	No	Yes
SLC16A7	1284	2831	No	No	No
MCOLN2	1231	1756	No	No	No
UNC119	1136	790	No	Yes	No
CXCR4	1095	975	No	No	Yes
MMP2	933	1417	No	No	Yes
ITPA	919	458	No	Yes	No
DNAJB12	914	2315	No	No	No
GPS2	907	229	No	No	No
BPIFA2A	900	833	No	No	Yes
KCNU1	896	2544	No	No	Yes
TRPC3	892	1303	No	Yes	No
ANKRD34C	867	3053	No	Yes	Yes
ILF2	866	126	No	No	Yes
CYP8B1	804	529	No	Yes	No
ASTE1	785	1292	No	Yes	No
YSK4	756	1742	No	No	No
PRSS21	719	37	No	Yes	No
RDH5	707	496	No	No	Yes
LSM10	649	349	No	No	Yes
DBC1	646	3393	No	No	No
SFXN1	643	1721	No	No	No
PTGER2	620	860	No	No	Yes
GPR89	615	1048	No	Yes	No
VIT	606	2722	No	No	No
FBXO6	586	499	No	No	No
B7H6	550	586	No	No	No

ADCY8	548	3841	No	No	No
PTPLAD2	535	885	No	No	Yes
BRPF3	517	1527	No	Yes	No
DHRS7C	515	406	No	No	Yes
RPL37	493	27	No	No	No
HTR1A	478	394	No	No	No
MLH1	472	1919	No	Yes	No
TMEM125	458	359	No	Yes	No
GPR110	446	1048	No	No	No
DHFR	438	490	No	Yes	No
RPL36AL	434	105	No	No	Yes
TLR5	423	513	No	Yes	No
BSPRY	421	550	No	No	Yes
BRB	418	206	No	No	Yes
EVI2B	417	440	No	No	Yes
ZNF648	416	361	No	No	No
NHLRC3	413	580	No	No	Yes
APPL2	411	812	No	Yes	No
COMMD1	406	1499	No	Yes	No
PRR5L	401	1260	No	No	Yes
KRT75	398	714	No	No	No
GPA33	392	960	No	No	No
C1H21orf62	390	515	No	No	Yes
PCDHB11	389	581	No	No	Yes
RASL10B	386	209	No	No	Yes
CHTF8	385	677	No	Yes	No
ATPBD4	382	950	No	Yes	No
CABS1	378	48	No	No	No
MAPK1	378	193	No	No	No
VGLL3	370	664	No	No	No
TOMM40L	368	626	No	No	No
CATSPER3	367	427	No	No	No
RPL6	363	6	No	No	No

*The intactness was considered if the reads were spread covering most of the exons

Supplemental Table S2: The tissue level expression (RNAs and proteins) and the reproductive functions of top 20 abundant transcripts in bovine spermatozoa.

Gene ID	Gene Name	Tissue Expression (RNA level)	Protein Localization	Reproductive Functions	References
PRM1	Protamine 1	Testis (enriched) and prostate	Nucleus and chromosome	Histone replacement and chromatin remodeling	1
CHMP5	Charged Multivesicular Body Protein 5	Testis and prostate	Cytoplasm, Cytoskeleton-Microtubules and Cytoskeleton-Cytokinetic bridge (Seminiferous duct)	Regulation of late endosome function during embryogenesis	2
YWHAZ	Tyrosine 3-Monooxygenase/Tryptophan 5-Monooxygenase Activation Protein, Zeta	Testis and Prostate	Intracellular (Cells in Seminiferous duct, Leydig cells and Glandular cells)	Spermatozoa protein phosphorylation	3
GTSF1L	Gametocyte Specific Factor 1-Like	Testis (enriched)	Intracellular (Cells in Seminiferous duct and Leydig cells)	Retrotransposon suppression of male germ cells during spermatogenesis	4
SCP2D1	SCP2 Sterol-Binding Domain Containing 1	Testis (enriched)	Intra cellular (Cells in Seminiferous duct and Leydig cells)	Positive association with semen quality	5
MGC137055	DET1 And DDB1 Associated 1 (uncharacterized protein)	Testis	Not available	Reported as full length transcript in bovine spermatozoa	6
CCDC181	Coiled-Coil Domain Containing 181	Testis (enriched)	Nucleoplasm (Seminiferous duct)	Involved in the microtubular manchette development of spermatozoa.	7
FABP1	Fatty Acid Binding Protein1	Not available	Not available	Spermatogenesis	8

THSD4	Thrombospondin Type 1 Domain Containing 4	Testis and Prostate (enriched)	Cells in Seminiferous duct, Leydig cells and Glandular cells	Not available	Not available
NR2E3	Nuclear Receptor Subfamily 2 Group E Member 3	Testis and Prostate	Nuclear receptor	mRNA expressed in testis	9
SV2C	Synaptic Vesicle Glycoprotein 2C	Testis and Prostate	Cells in Seminiferous duct, Leydig cells and Glandular cells	Not available	Not available
TOE1	Target of EGR1, Membrane 1 (Nuclear)	Testis and prostate	Cells in Seminiferous duct, Leydig cells and Glandular cells	Not available	Not available
SLC16A7	Solute Carrier Family 16 Member 7	Testis and prostate	Cells in Seminiferous ducts	Involved in sperm metabolism	10
MCOLN2	Mucolipin 2	Testis and prostate	Cells in Seminiferous duct, Leydig cells and Glandular cells	Expressed in epididymis	11
UNC119	Unc-119 Lipid Binding Chaperone	Testis and prostate	Glandular cells	Not available	Not available
CXCR4	C-X-C Motif Chemokine Receptor 4	Testis and prostate	Cells in seminiferous ducts and Glandular cells	Chemokine signaling molecule in spermatozoa	12
PAG5	Pregnancy associated glycoprotein 5	Testis and prostate	Cells in seminiferous ducts, Leydig cells and Glandular cells	Reported in bovine placenta	13
MMP2	Matrix Metallopeptidase 2 (Gelatinase A, 72Kda Gelatinase, 72kDa type IV Collagenase)	Testis and prostate	Leydig cells and Glandular cells	Penetration of zona pellucida	14
ITPA	Inosine	Testis and	Cells in	mRNA	15

	Triphosphatase (Nucleoside Triphosphate Pyrophosphatase)	prostate	seminiferous ducts, Leydig cells and Glandular cells	expression was high in testis.	
DNAJB12	DnaJ Heat Shock Protein Family (Hsp40) Member B12	Testis and prostate	Cells in seminiferous ducts and Glandular cells	Chaperone (Similar to HSP40)	16

Note: The above information's are obtained from www.ncbi.nlm.nih.gov/pubmed, www. gopubmed.org; www.ebi.ac.uk, www.proteinatlas.org, and www.uniprot.org

Supplemental Table S3: The abundantly expressed (Top 10) LOCs in the bovine spermatozoa.

Gene ID	Gene Name/Description	TPM	UGR
LOC100335847	Protamine 1-like	7817	231
LOC786921	Deubiquitinating enzyme 3-like	6418	1610
LOC101906132	--	4391	711
LOC404073	Histone H2B variant PT15	4174	175
LOC786597	Chromosome 20 open reading frame 106 homolog	3887	244
LOC100335236	PREDICTED: Bos taurus hypothetical protein LOC100335236 transcript	3742	2374
LOC786327	T-cell activation Rho GTPase-activating protein-like	3571	207
LOC786899	T-cell activation Rho GTPase-activating protein-like	3379	1253
LOC781191	Uncharacterized protein, found in RNA derived from captured sperm of mixed chromosome content. The gene mapping by the National Center for Biotechnology Information (NCBI) located DNA for GX1 to the bovine X chromosome (NCBI, U.S. National Library of Medicine, 8600 Rockville Pike, Bethesda, Md. 20894; Gene ID 781191).	3283	27
LOC101905684	--	2495	149

Supplemental Table S4: The abundantly expressed (Top 20) non-coding RNAs in the bovine spermatozoa with unique gene reads.

Transcript ID	TPM	Unique Gene Reads
NONBTAT002138.2	52526	361
NONBTAT029740.1	46868	3315
NONBTAT026069.2	39900	2036
NONBTAT026075.2	26794	2320
NONBTAT015718.2	14800	259
NONBTAT015717.2	13829	206
NONBTAT017665.2	13513	205
NONBTAT027794.1	7933	2971
NONBTAT007060.2	7921	29
NONBTAT029554.1	7846	6267
NONBTAT008960.2	7804	6206
NONBTAT022624.2	6306	3723
NONBTAT030080.1	6070	1454
NONBTAT030770.1	5672	8489
NONBTAT030342.1	4966	5823
NONBTAT030589.1	4619	249
NONBTAT031209.1	4369	4505
NONBTAT022014.2	4324	6851
NONBTAT025041.2	4239	205
NONBTAT001981.2	4089	557

Supplemental Table S5: The pathways associated with spermatozoal RNAs. The analysis was carried out using transcripts with more than 100 TPM with *Bos taurus* as background using DAVID bioinformatics software.

KEEG Pathway	P-value	Genes Count	Observed Genes ID
Ribosome	4.96E-09	23	*RPL27A, RPL27, RPL37, RPS2, RPS5, RPL28, RPS3, RPS18, RPS27, RPL32, RPL23, RPL18A, RPS29, RPL31, RPL6, RPL13A, RPLP0, RPS14, FAU, RPL10A, RPS11, UBA52, RPS23*
Axon guidance	0.01	12	*PAK6, MAPK1, EPHA7, EPHB6, PLXNA4, CXCR4, SEMA4F, SEMA3E, CFL1, SEMA3D, UNC5D, ARHGEF12*
Salmonella infection	0.01	9	*ACTB, FOS, PFN1, MAPK1, CCL3, IL18, IL1B, TLR5, FLNB*
Viral myocarditis	0.01	8	*ACTB, EIF4G2, PRF1, EIF4G3, BOLA-DOA, BLA-DQB, DAG1, SGCD*
Inflammatory bowel disease (IBD)	0.02	8	*IL12RB2, STAT4, IL18, BOLA-DOA, BLA-DQB, IL1B, TLR5, STAT1*
PPAR signaling pathway	0.02	8	*SLC27A1, GK2, UBC, FABP3, ACSBG2, RXRG, FABP1, CYP8B1*
Galactose metabolism	0.03	5	*PFKL, AKR1B1, PFKP, G6PC3, G6PC2*
Glutathione metabolism	0.05	6	*GPX2, GSTM3, SRM, GSTO1, GCLM*
Oocyte meiosis	0.07	9	*PLCZ1, IGF1R, MAPK1, YWHAZ, ADCY8, ANAPC13, SKP1, CDC25C, CALM2*
RNA degradation	0.07	7	*PFKL, BTG1, PFKP, RQCD1, DHX36, LSM2, CNOT6*

Supplemental Table S6: Cluster analysis of commonly expressed genes in all animals detected by Ion Proton and Illumina platforms. These genes considered for this analysis were not differentially expressed between platforms.

Category	Term	Genes Count	P-value
**Annotation Cluster 1**	**Enrichment Score: 2.60**		
UP_KEYWORDS	ATP-binding	37	0.00
GOTERM_MF_DIRECT	GO:0005524~ATP binding	22	0.00
UP_KEYWORDS	Nucleotide-binding	43	0.01
**Annotation Cluster 2**	**Enrichment Score: 1.60**		
INTERPRO	IPR019844:Cold-shock conserved site	3	0.01
INTERPRO	IPR002059:Cold-shock protein, DNA-binding	3	0.02
INTERPRO	IPR011129:Cold shock protein	3	0.02
SMART	SM00357:SM00357	3	0.02
INTERPRO	IPR012340:Nucleic acid-binding, OB-fold	5	0.11
**Annotation Cluster 3**	**Enrichment Score: 1.58**		
UP_KEYWORDS	Zinc-finger	31	0.01
UP_KEYWORDS	Metal-binding	60	0.03
UP_KEYWORDS	Zinc	38	0.05
**Annotation Cluster 4**	**Enrichment Score: 1.46**		
UP_KEYWORDS	TPR repeat	8	0.00
INTERPRO	IPR011990:Tetratricopeptide-like helical	11	0.01
UP_SEQ_FEATURE	repeat:TPR 2	4	0.07
UP_SEQ_FEATURE	repeat:TPR 1	4	0.07
UP_SEQ_FEATURE	repeat:TPR 3	3	0.19
**Annotation Cluster 5**	**Enrichment Score: 1.46**		
UP_KEYWORDS	TPR repeat	8	0.00
INTERPRO	IPR011990:Tetratricopeptide-like helical	11	0.01
INTERPRO	IPR013026:Tetratricopeptide repeat-containing domain	8	0.02
INTERPRO	IPR019734:Tetratricopeptide repeat	7	0.05
INTERPRO	IPR013105:Tetratricopeptide TPR2	3	0.20
SMART	SM00028:SM00028	5	0.20
**Annotation Cluster 6**	**Enrichment Score: 1.37**		
UP_KEYWORDS	Microtubule	11	0.00
GOTERM_BP_DIRECT	GO:0007017~microtubule-based process	3	0.06

GOTERM_CC_DIRECT	GO:0005874~microtubule	6	0.08
GOTERM_BP_DIRECT	GO:0008152~metabolic process	8	0.26
**Annotation Cluster 7**	**Enrichment Score: 1.36**		
KEGG_PATHWAY	bta03040:Spliceosome	12	0.00
INTERPRO	IPR013126:Heat shock protein 70 family	4	0.01
INTERPRO	IPR018181:Heat shock protein 70, conserved site	4	0.01
KEGG_PATHWAY	bta04915:Estrogen signaling pathway	9	0.01
KEGG_PATHWAY	bta05169:Epstein-Barr virus infection	13	0.01
KEGG_PATHWAY	bta05134:Legionellosis	6	0.03
KEGG_PATHWAY	bta04141:Protein processing in endoplasmic reticulum	10	0.06
UP_KEYWORDS	Stress response	5	0.07
KEGG_PATHWAY	bta04010:MAPK signaling pathway	13	0.09
KEGG_PATHWAY	bta05145:Toxoplasmosis	7	0.16
KEGG_PATHWAY	bta04612:Antigen processing and presentation	5	0.19
KEGG_PATHWAY	bta05164:Influenza A	8	0.27
UP_KEYWORDS	Chaperone	6	0.28
KEGG_PATHWAY	bta05162:Measles	6	0.41
**Annotation Cluster 8**	**Enrichment Score: 1.07**		
KEGG_PATHWAY	bta05231:Choline metabolism in cancer	8	0.03
KEGG_PATHWAY	bta04666:Fc gamma R-mediated phagocytosis	7	0.04
KEGG_PATHWAY	bta05132:Salmonella infection	4	0.46
**Annotation Cluster 9**	**Enrichment Score: 0.97**		
INTERPRO	IPR019821:Kinesin, motor region, conserved site	3	0.09
INTERPRO	IPR001752:Kinesin, motor domain	3	0.10
UP_KEYWORDS	Motor protein	5	0.10
SMART	SM00129:SM00129	3	0.13
**Annotation Cluster 10**	**Enrichment Score: 0.96**		
GOTERM_MF_DIRECT	GO:0005516~calmodulin binding	5	0.05
UP_KEYWORDS	Calmodulin-binding	5	0.05
KEGG_PATHWAY	bta04020:Calcium signaling pathway	7	0.49

Supplemental Table S7: The primers used for assessing the contamination of epithelial cells, germ cells and leukocytes RNA and also spermatozoal gDNA in isolated spermatozoal total RNA.

Primer ID	Primer Sequence (5' – 3')	Primer length (bp)	Product size (bp)	Intron Span (Yes/No)	Accession Number	Purpose
CDH1-F	CTGCATTCCTGGCTTTGGTG	20	171	Yes	NM_001002763.1	Epithelial cell
CDH1-R	GTAAGCACGCCATCTGTGTG	20				
KIT- F	GAATAGCTGGCATCAGGGTG	20	224	Yes	AF263827.1	Germ cell
KIT- R	CCAGATCCACATTCTCTCCATC	22				
PTPRC- F	ACCCAACCTTCTACTCAAGATG	22	124	Yes	NM_001206523.1	Leukocytes
PTPRC- R	CGTATTTGTTCTCACATGGTGG	22				
PRM1- F	AAGATGTCGCAGACGAAGGAG	21	222	Yes	NM_174156.2	Sperm RNA & gDNA
PRM1- R	GTGGCATTGTTCGTTAGCAGG	21				
PRM1- F*	ATGGCCAGATACCGATGC	18	156	Yes	NM_174156.2	Coding region
PRM1- R*	TTACTGTCTTGTACACCT	18				

* The protamine1 (PRM1) primer covering coding region was used for confirmation of presence of spermatozoal RNA.

Supplemental Table S8: The primers used for validating transcripts presence in spermatozoa. The transcripts were selected based on the expression levels (TPM) and read coverage.

Primer ID	Primer sequence (5' – 3')	Primer length (bp)	Product size (bp)	Intron Span Yes/No	Accession Number
HMGB4-F	AGACGGATGTGGACAAGCAG	20	150	No	NM_001040562.3
HMGB4-R	GCTCGAACTCTCTGCACTGG	20			
CHMP5-F	TGACCTCGAAGCAGAGTTGG	20	120	Yes/E-E	NM_001034682.2
CHMP5-R	TTCGTGTCAGTGGGAACACC	20			
YWHAZ-F	GGAAATGCAACCAACACATCC	21	126	Yes/E-E	NM_174814.2
YWHAZ-R	ATGGCTTCATCAAATGCTGTC	21			
SCP2D1-F	AGTGCAGTGGGATGTGGAAG	20	133	No	NM_001040507.2
SCP2D1-R	TCTGACAGCGAGAGAGGGTG	20			
GTSF1L-F	GGCATCTGCTTTGTGTGGTC	20	137	No	NM_001079601.2
GTSF1l-R	ACAACTCGGGCTGCCTATTC	20			
C23H6orf62-F	AGACAGCACTTGCAGACTCC	20	134	Yes/E-E	NM_001077999.2
C23H6orf62-R	GGCATGTAGGGATGGAGGTG	20			
SDCBP2-F	TCCAGAAAGTGCCAGTGCAG	20	126	No	XM_003586805.2
SDCBP2-R	CATAGGTGGATCTCGCGCAC	20			
HSFY2-F	AAGCAAGTGTCAACGAAGATG	21	128	No	NM_001077006.1
HSFY2-R	GACAATCTGGATGGTATGAGGC	22			
OR6C2-F	GCTGCTTGTGCCAGTCAAC	19	149	No	XM_001254810.2
OR6C2-R	ACGTTCTTGCAGACTCTAATGC	22			
MGC157405-F	TACACATGGACCGCATCTCC	20	135	Yes/E-E	NM_001083697.2
MGC157405-R	CGCCGATGAGCTTCTGTATG	20			
ZNF280B-F	GGGACCGGATGTGACCTTC	19	103	Yes/E-E	NM_001077935.1
ZNF280B-R	CCACAAGTCCCAGTGCTTCC	20			
PLCZ1-F	TGCATGAACAGAGGTTACCG	20	118	No	NM_001011680.2
PLCZ1-R	TGTCAAGTACCATGAGTGGC	20			

Supplemental Figure 1: The work flow of spermatozoal RNA sequencing procedure and the softwares used for analyzing the transcriptome data.

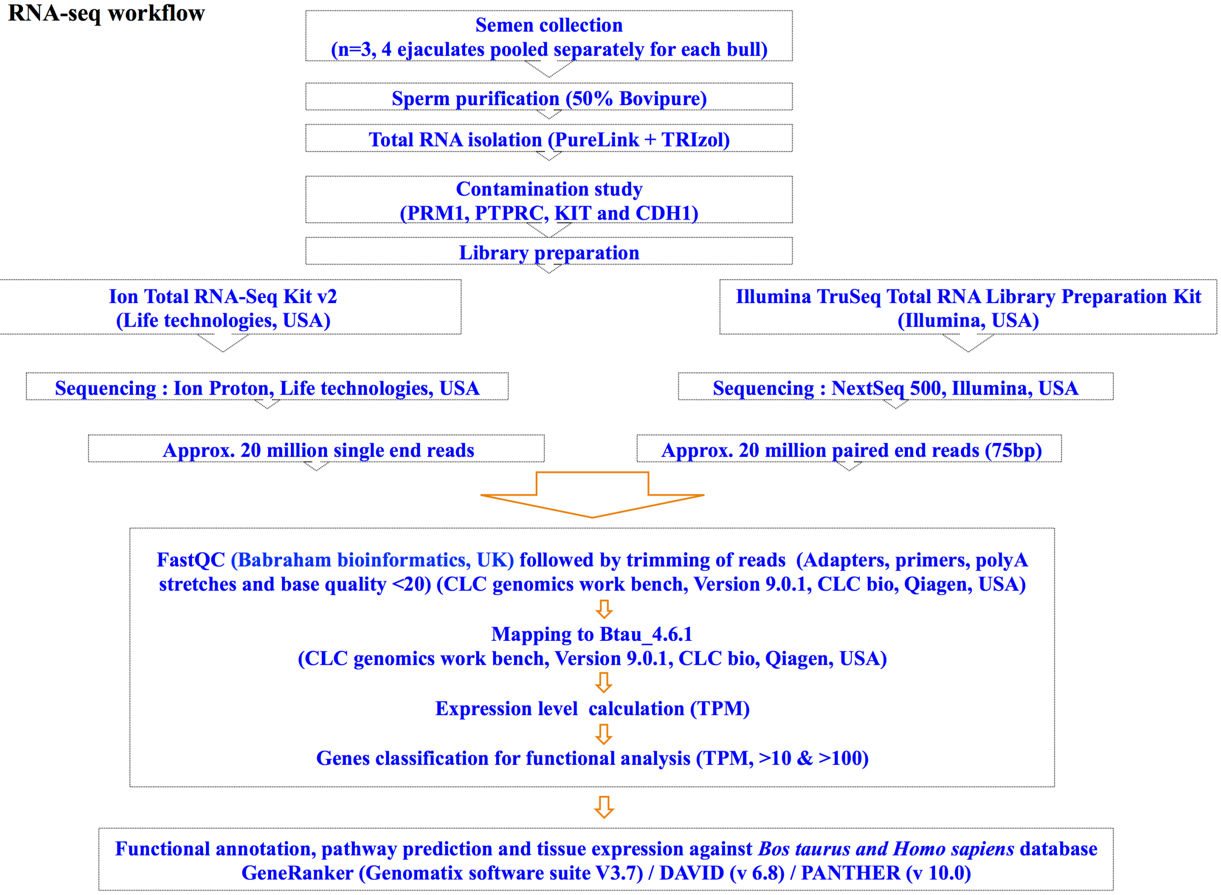

Supplemental Figure 2: The involvement of abundant (>100 TPM) spermatozoal transcripts in axon guidance (a) and oocyte meiosis (b) based on KEGG pathway (http://www.kegg.jp/kegg/kegg2.html). The genes observed in the present study were highlighted in red colour.

a) Axon guidance

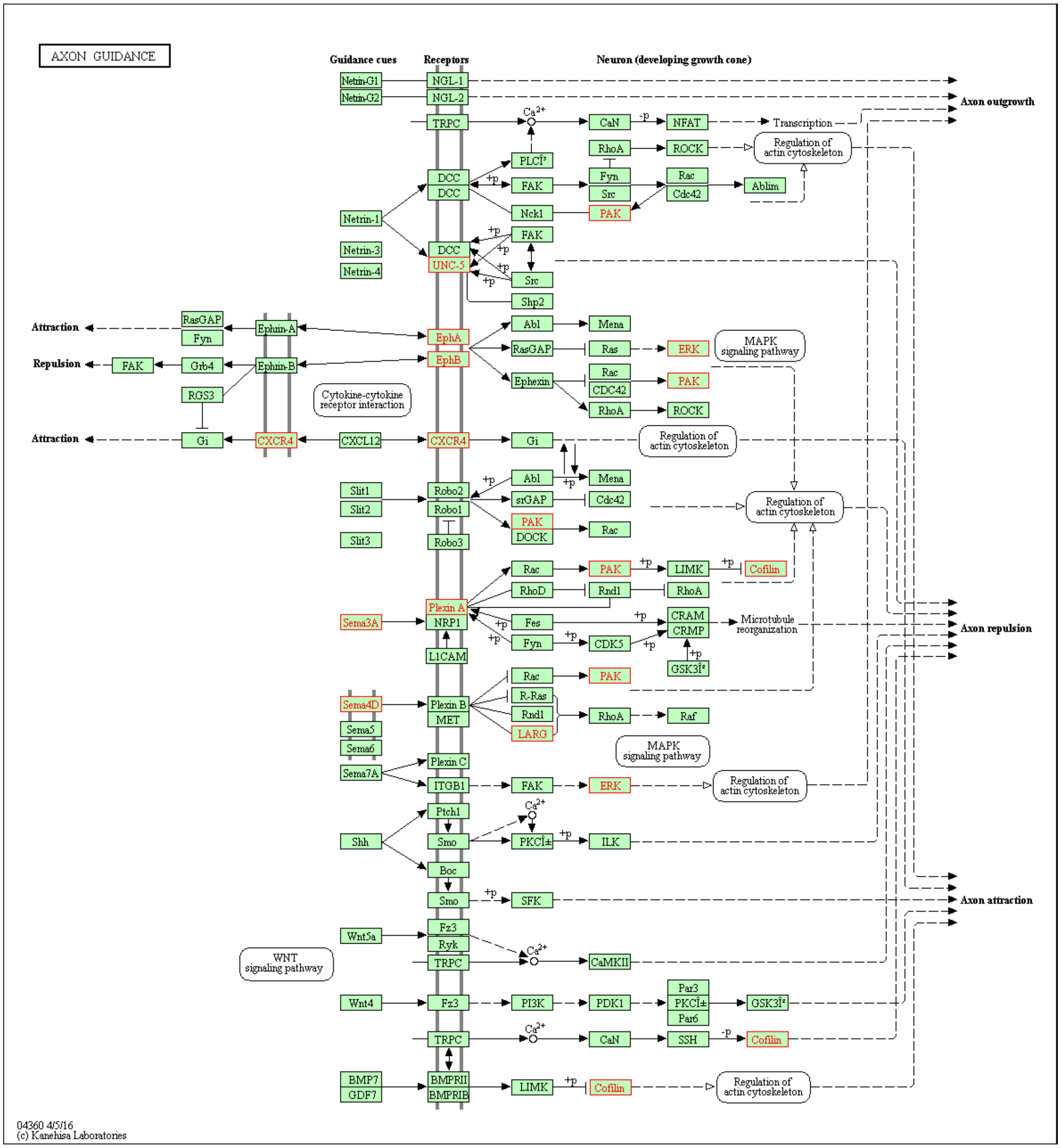

b) Oocyte meiosis

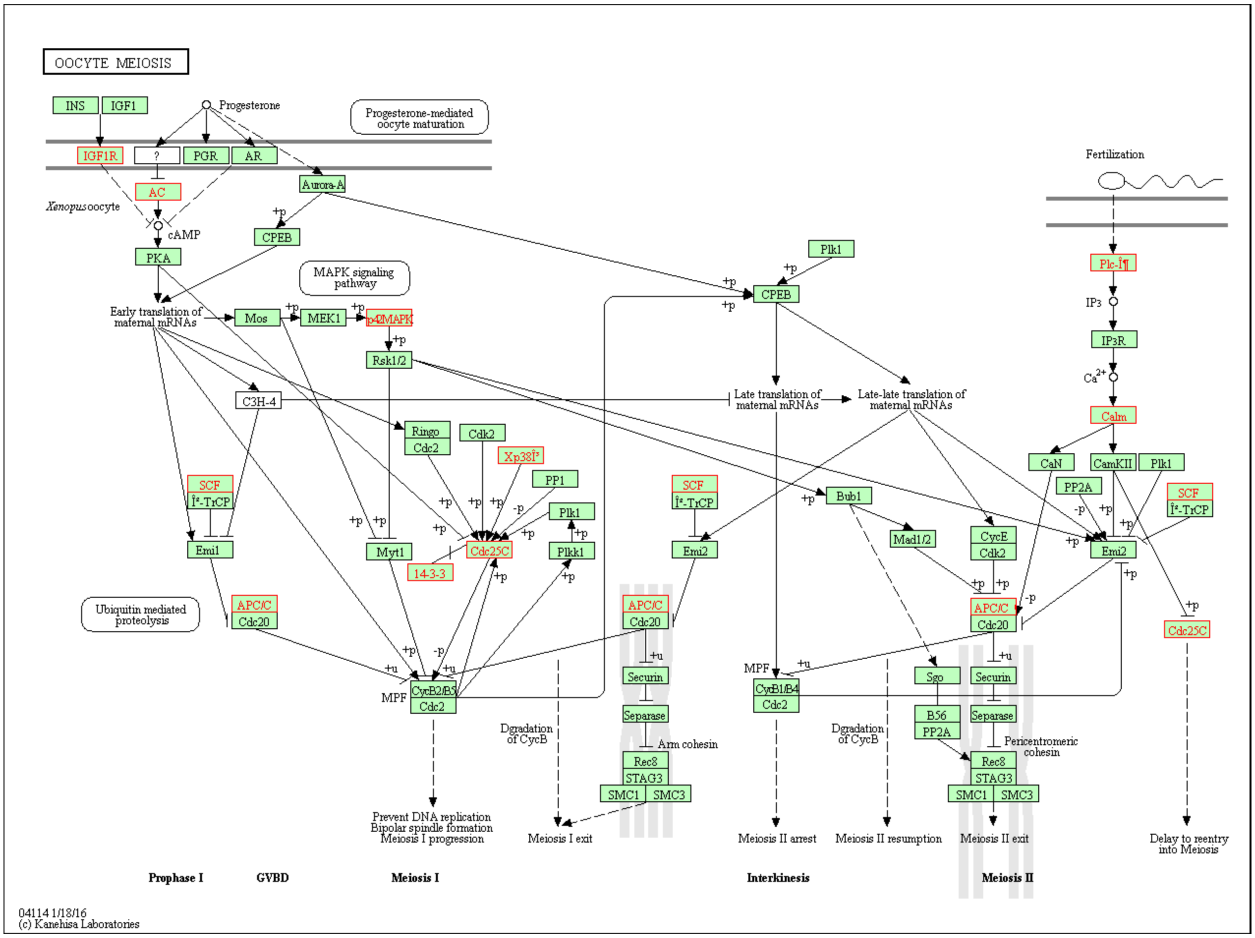

Supplemental Figure 3: The common number of genes (top 328 transcripts) observed in this current study from fresh bull spermatozoa were compared with the earlier study in cryopreserved sperm[6].

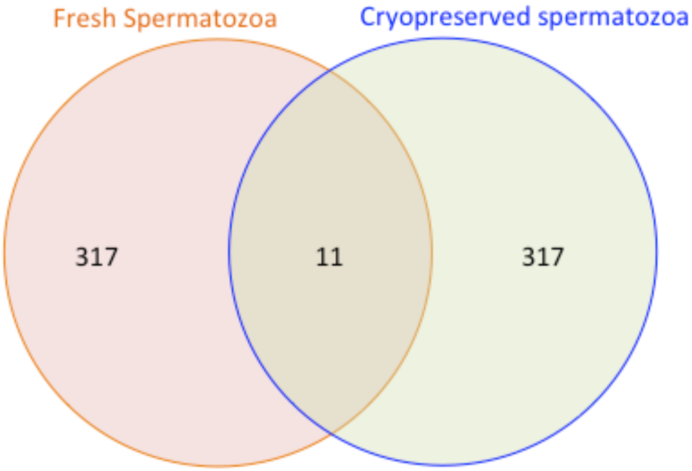

References

1. Dogan, S. *et al.* Sperm protamine-status correlates to the fertility of breeding bulls. *Biol. Reprod.* **92,** 1-9 (2015).

2. Shim, J. H. *et al.* CHMP5 is essential for late endosome function and down-regulation of receptor signaling during mouse embryogenesis. *J. Cell Biol*. **172,**1045-1056 (2006).

3. Puri, P., Myers, K., Kline, D. & Vijayaraghavan, S. Proteomic analysis of bovine sperm YWHA binding partners identify proteins involved in signaling and metabolism. *Biol. Reprod.* **79,** 1183-1191(2008).

4. Yoshimura, T. *et al.* Gtsf1/Cue110, a gene encoding a protein with two copies of a CHHC Zn-finger motif, is involved in spermatogenesis and retrotransposon suppression in murine testes. *Develop. Biol.* **335,** 216-227 (2009).

5. Ing, N. H. *et al.* Sequencing of RNA from stallion sperm identifies potential markers of fertility. *J. Equine Vet. Sci.* **43,** S64 (2016).

6. Card, C. J. et al. Cryopreserved bovine spermatozoal transcript profile as revealed by high-throughput ribonucleic acid sequencing. *Biol. Reprod.* **88,** 49 (2013).

7. Schwarz, T., Prieler, B., Schmid, J. & Neesen, J. Molecular characterization of Ccdc181, a Hook1 and microtubule interacting protein. 11th YSA PhD Symposium, Abstract Book. Abstract no. 112. 72-73 (2015).

8. Liu, R. Z., Li, X. & Godbout, R. A novel fatty acid-binding protein (FABP) gene resulting from tandem gene duplication in mammals: transcription in rat retina and testis. *Genomics,* **92,** 436-445 (2008).

9. Nishimura, M., Naito, S. & Yokoi, T. Tissue-specific mRNA expression profiles of human nuclear receptor subfamilies. *Drug Metab. Pharmacokinet.* **19,** 135-149 (2004).

10. Lee, J., Lee, D. R. & Lee, S. The genetic variation in Monocarboxylic acid transporter 2 (MCT2) has functional and clinical relevance with male infertility. *Asian J. Androl.* **16,** 694 (2014).

11. Zhang, J. S., *et al.* Genome-wide profiling of segmental-regulated transcriptomes in human epididymis using oligo microarray. *Mol. Cell. Endocrinol.* **250,** 169-177 (2006).

12. Zuccarello, D. *et al.* How the human spermatozoa sense the oocyte: a new role of SDF1-CXCR4 signalling. *Int. J. Androl. **34,*** e554-e565 (2011).

13. Ushizawa, K. *et al.* Global gene expression analysis and regulation of the principal genes expressed in bovine placenta in relation to the transcription factor AP-2 family. *Reprod. Biol. Endocrinol.* **5,** 17 (2007).

14. Ferrer, M. *et al.* MMP2 and acrosin are major proteinases associated with the inner acrosomal membrane and may cooperate in sperm penetration of the zona pellucida during fertilization. *Cell Tissue Res.* **349,** 881-895 (2012).

15. Behmanesh, M. *et al.* Characterization of the structure and expression of mouse Itpa gene and its related sequences in the mouse genome. *DNA Res.* **12,** 39-51(2005).

16. Wenhao, T. *et al.* miRNA-mediated regulation of heat shock proteins in human ejaculated spermatozoa. *Turkish J. Med. Sci.* **45,** 1285-1291(2015).
